# KRAS mutation: from undruggable to druggable in cancer

**DOI:** 10.1038/s41392-021-00780-4

**Published:** 2021-11-15

**Authors:** Lamei Huang, Zhixing Guo, Fang Wang, Liwu Fu

**Affiliations:** State Key Laboratory of Oncology in South China; Collaborative Innovation Center for Cancer Medicine; Guangdong Esophageal Cancer Institute, Sun Yat-Sen University Cancer Center, Guangzhou, 510060 P. R. China

**Keywords:** Oncogenes, Cancer therapy, Drug development, Target identification

## Abstract

Cancer is the leading cause of death worldwide, and its treatment and outcomes have been dramatically revolutionised by targeted therapies. As the most frequently mutated oncogene, Kirsten rat sarcoma viral oncogene homologue (KRAS) has attracted substantial attention. The understanding of KRAS is constantly being updated by numerous studies on KRAS in the initiation and progression of cancer diseases. However, KRAS has been deemed a challenging therapeutic target, even “undruggable”, after drug-targeting efforts over the past four decades. Recently, there have been surprising advances in directly targeted drugs for KRAS, especially in KRAS (G12C) inhibitors, such as AMG510 (sotorasib) and MRTX849 (adagrasib), which have obtained encouraging results in clinical trials. Excitingly, AMG510 was the first drug-targeting KRAS (G12C) to be approved for clinical use this year. This review summarises the most recent understanding of fundamental aspects of KRAS, the relationship between the KRAS mutations and tumour immune evasion, and new progress in targeting KRAS, particularly KRAS (G12C). Moreover, the possible mechanisms of resistance to KRAS (G12C) inhibitors and possible combination therapies are summarised, with a view to providing the best regimen for individualised treatment with KRAS (G12C) inhibitors and achieving truly precise treatment.

## Introduction

Kirsten rat sarcoma viral oncogene homologue (KRAS) is the best-known oncogene with the highest mutation rate among all cancers and is associated with a series of highly fatal cancers, including pancreatic ductal adenocarcinoma (PDAC), nonsmall-cell lung cancer (NSCLC), and colorectal cancer (CRC). The identification of tumour driver genes and the development of specific inhibitors have revolutionised cancer treatment strategies and clinical outcomes. Numerous clinical results have shown that targeted therapies significantly extend progression-free survival and are less toxic than standard chemotherapy^1–3^. For instance, targeted therapies in patients harbouring epidermal growth factor receptor (EGFR)-sensitive mutation or anaplastic lymphoma kinase (ALK) gene fusion have markedly enhanced survival time, with a median overall survival of 3 years or more^4–6^. Unfortunately, despite 40 years of proprietary drug efforts, there are still no effective strategies targeting KRAS mutations, except for sotorasib, which has just been approved to target the mutated KRAS subtype KRAS (G12C). Due to the intrinsic characteristics of KRAS proteins, targeting KRAS has been considered to be quite challenging. Therefore, many efforts have focused on indirectly targeting KRAS, including targeting its downstream signalling effectors^7^, epigenetic approaches such as telomerase inhibitors^8^ and RNA interference^9^ and synthetic lethality approaches, such as cyclin-dependent kinase inhibitors^10^. However, most of these strategies have failed due to a lack of activity or selectivity. In addition, patients with KRAS mutations usually have a poor response to current standard therapy^11^. There has been an urgent and unmet need to target KRAS mutations in KRAS-driven cancer. Recently, there has been light on the horizon for one specific mutation, KRAS (G12C). With the discovery of a new allosteric site of KRAS (G12C), several irreversible covalently binding inhibitors of KRAS (G12C) have emerged, raising the hope of drugging KRAS. In this review, the latest research progress on KRAS, the characteristics of KRAS mutations, the relationship between the KRAS mutations and tumour immunity, and the targeted research progress of KRAS, especially KRAS (G12C), are summarised. Furthermore, the potential mechanisms of resistance to KRAS (G12C) inhibitors and promising combination strategies to determine the best-individualised treatment regimen are outlined.

## KRAS and signal transduction

### Introduction to KRAS

**Fig. 1:**
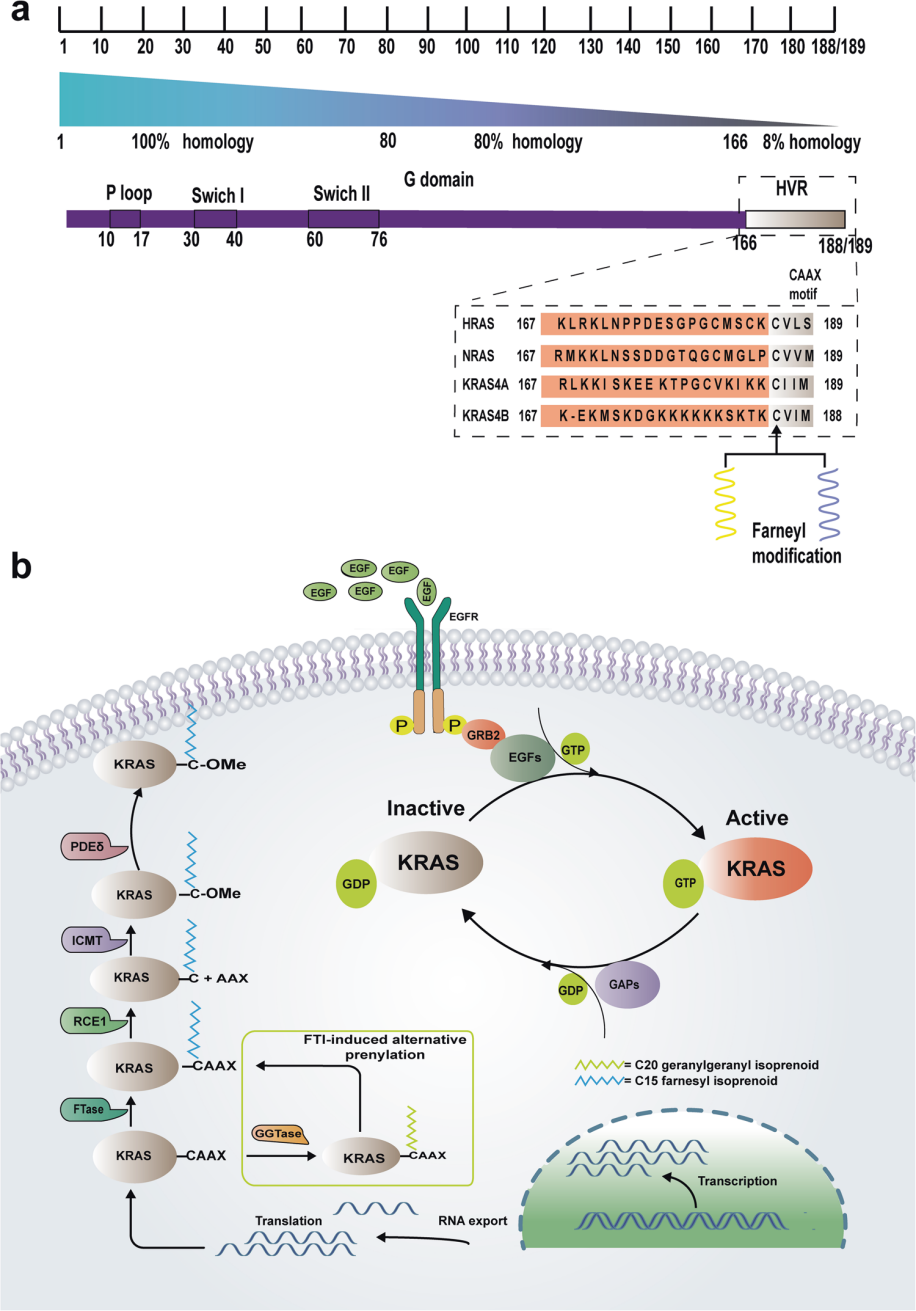
The structure and function of KRAS. **a** According to homology, KRAS, which consists of 188/189 amino acids can be divided into three parts. The first part consisting of the first 85 amino acid residues is a highly conserved region. The next 80 amino acid residues are defined as a second part where homology between any pair of human RAS genes is 85%. A third part is a highly variable region and homology is only 8%. KRAS forms two major domains: a catalytic domain called the G domain and a hypervariable region (HVR). The G domain consists of three regions: switch I, switch II and the P loop, which binds guanine nucleotides and activates signalling pathway by interacting with effectors. The HVR consists of a membrane-targeting domain containing the CAAX motif where C is a cysteine, A is any aliphatic amino acid and X is any amide acid, which acquires lipids by farnesyl or prenyl modification. **b** The normal function of KRAS depends on the membrane localisation of its post-transcriptional modification, which is mediated by a series of enzymes. KRAS functions as a guanosine diphosphate (GDP)/triphosphate (GTP) binary switch, which controls important signal transduction from activated membrane receptors to intracellular molecules. The binary switch is mainly determined by two kinds of regulatory proteins: guanine nucleotide exchange factors (GEFs) and GTPase-activating proteins (GAPs). FTase: farnesyltransferase; GGTase: geranyl geranyltransferase; RCE1: RAS-converting enzyme 1; ICMT: isoprenylcysteine carboxyl methyltransferase; PDEδ: phosphodiesterase δ

The KRAS gene is a member of the rat sarcoma viral oncogene family (RAS), which includes two other isoforms in humans: the Harvey and neuroblastoma rat sarcoma viral oncogenes (HRAS, NRAS). In 1982, Weinberg and Barbacid isolated a gene from human bladder cancer cell lines. Subsequently, this gene was identified as a human homologue of the RAS gene, named HRAS, located on the short arm of chromosome 11 (11p15.1–11p15.3)^12^. In the same year, another homologue was found in human lung cancer cells, called KRAS, located on the short arm of chromosome 12 (12p11.1–12p12.1). The last gene, called NRAS, is found in human neuroblastoma and is located on the short arm of chromosome 1 (1p22–1p32)^13–15^. RAS genes are evolutionarily conserved with similar structures and are composed of four exons distributed on the full length of approximately 30 kb DNA. The KRAS gene encodes two highly related protein isoforms, KRAS-4B and KRAS-4A, which consist of 188 and 189 amino acids, respectively, due to different clipping of the fourth exon^16^. The other two RAS proteins all contain 189 amino acids. The term KRAS is generally referred to as KRAS-4B due to the high level of mRNA encoding KRAS-4B in cells^17^. The crystal structure of RAS reveals six beta strands and five alpha helices^18^. which form two major domains: a catalytic domain called the G domain and a hypervariable region (HVR)^19^. The G domain consists of three regions: switch I, switch II, and the P loop, which binds guanine nucleotides and activates signalling by interacting with effectors. The HVR comprises the CAAX motif related to membrane localisation^20^. From the perspective of function, RAS is a kind of membrane-bound regulatory protein (G protein) binding guanine nucleotide belonging to the family of guanosine triphosphatases (GTPases)^21^. RAS functions as a guanosine diphosphate (GDP)/triphosphate (GTP) binary switch, which controls important signal transduction from activated membrane receptors to intracellular molecules^22^ The binary switch is mainly determined by two kinds of regulatory proteins: guanine nucleotide exchange factors (GEFs) such as son of sevenless (SOS) and GTPase-activating proteins (GAPs) such as neurofibromin 1 (NF1)^23^. In the resting state, KRAS normally binds with GDP in an inactivated state due to the intrinsic GTPase activity of KRAS, which is able to hydrolyse GTP to GDP^24^. When the cells receive the relevant stimuli, such as the interaction of EGF and EGFR, the KRAS-GDP complex appears to have a decreased affinity of KRAS with GDP in the presence of GEFs, and then GDP is replaced by GTP, which has a higher affinity and an approximately 10-fold higher cellular concentration than GDP^25^. KRAS-GTP binding acquires an altered conformation in switches I and II of the G domain, and then KRAS is activated and binds to its downstream molecules as a monomer or dimer to mediate a series of signalling cascades. In contrast, GAPs promote the binding between GDP and KRAS by enhancing the GTPase activity of KRAS, thus maintaining the inactive state of KRAS (Fig. 1)^26^.

### KRAS signalling

KRAS proteins function as a finely regulated molecular switch that controls multiple signalling cascades by cycling between activated and inactivated conformations. KRAS proteins can be activated by growth factors, chemokines, Ca^2+^ or receptor tyrosine kinase (RTK). Activated KRAS protein can activate multiple signalling pathways, including the rapidly accelerated fibrosarcoma (RAF)-mitogen-activated protein kinase (MEK)-extracellular regulated protein kinases (ERK) signalling pathway, phosphoinositide 3-kinase (PI3K)-protein kinase B (AKT)—mammalian target of rapamycin (mTOR) signalling pathway, and other signalling pathways, revealing a wide range of KRAS communications with multiple signalling pathways.

### The upstream regulation of KRAS

#### GRB2-SOS1 complex

Many growth factors, such as epidermal growth factor (EGF), platelet-derived growth factor (PDGF) and fibroblast growth factors (FGFs), activate receptor tyrosine kinases (RTKs) and then activate KRAS proteins through intermediary molecules. A typical example is the interaction of EGF with EGFR, which results in the dimerisation and cross-phosphorylation of EGFR^27^. Phosphorylated EGFR is bound by growth factor receptor-bound protein 2 (GRB2), an adaptor molecule, through their respective SH2 domains. GRB2 consists of one SH2 domain and two SH3 domains, of which the SH3 domain is capable of binding SOS1, a kind of GEF. Activated SOS1 promotes the binding of GTP and KRAS and converts KRAS from an inactive state to an active state. As mentioned earlier, KRAS states are tuned primarily to GEFs/GAPs. Therefore, molecules upstream of KRAS mainly mediate the activation or inactivation of KRAS by regulating these two types of molecules. The signalling cascades mediated by the KRAS–GTP complex were thought to occur only within the cell membrane in the past. Recently, Tulpule and his colleagues revealed another novel, membrane-independent approach to activate KRAS^28^. Some fusion proteins containing RTKs, such as EML4-ALK, can combine with GRB2 and SOS to form membraneless cytoplasmic protein granules to actively activate KRAS and activate downstream signals. Notably, this approach is similar but not identical to phase separation.

#### RAS-GRF1

RAS protein-specific guanine nucleotide releasing factor 1 (RAS-GRF1) is another GEF and is expressed primarily in the brain. In mature neurons, RAS-GRF1 connects signals from glutamate receptors, such as N-methyl-D-aspartic acid receptor (NMDAR) to KRAS, thereby promoting the downstream MAPK/ERK cascade reaction^29^. The ability of RAS-GRF1 to activate KRAS was significantly enhanced with increasing Ca^2+^ concentration, revealing the internal communication and mutual regulation between KRAS signalling and Ca^2+^ signalling as well as metabolism^30,31^. In addition, chemokines can activate protein kinase A by producing cAMP after binding to chemokine receptors on the cell membrane. Activated protein kinase A can activate RAS-GRF1 through phosphorylation, thereby promoting the transformation of KRAS into an active state^32^.

#### SHP2

In addition to the GEFs mentioned above as a key molecule in KRAS activation, Src homology phosphatase 2 **(**SHP2) plays an integral role in KRAS activation^33,34^. SHP2 is a protein tyrosine phosphatase (PTP) containing Src homology 2 (SH2) domains and is encoded by PTPN11^35^. Unlike most PTPs, numerous studies indicate that SHP2 functions in the activation of intracellular signalling pathways, particularly the KRAS/ERK pathway^34^. SHP2 is a common signalling button that mediates numerous receptor tyrosine kinase signals to KRAS-ERK signalling^36^. On the one hand, SHP2 can promote the recruitment of the GRB2-SOS1 compound to the receptor. Studies have identified some phosphorylation sites of SHP2 as the major binding site for GRB2, such as tyrosine 542 and 580 of SHP2^37^. SHP2 therefore acts as a scaffolding effect in GRB2 recruitment. On the other hand, SHP2 catalytic activity was required to promote the activation of KRAS through the dephosphorylation of the SHP2 substrate^38^. Some dephosphorylation substrates of SHP-2 have been shown to promote KRAS activation. There are also many negative regulatory molecules involved in KRAS activation, such as p120-RASGAP, a GTPase-activating protein. RASGAP has been shown to be a target for SHP2. SHP2 dephosphorylates the p120-Rasgap junction and regulates the recruitment of p120-RASGAP near KRAS, thus relieving the negative regulatory effect of p120-RASGAP^39,40^. In addition to p120-RASGAP, SHP2 plays a signalling role by dephosphorylating other molecules, including negative regulators of Sprouty and activators of Src, through the dephosphorylation of Src-regulatory proteins^41,42^.

### The KRAS-mediated signalling pathway

#### The RAF-MEK-ERK pathway

The RAF-MEK-ERK pathway is the canonical downstream target of KRAS signalling^43^. Activated KRAS-GTP can recruit rapidly accelerating fibrosarcoma (RAF), a serine/threonine-specific protein kinase, from the cytoplasm to the plasma membrane, induce conformational changes in RAF and promote the activation of RAF by homologous or heterologous dimerization^44^. The C-terminal catalytic domain of RAF binds to MEK1/2 and activates it by phosphorylation. MEK1/2 phosphorylates and activates ERK1/2, and activated ERK phosphorylates ribosomal S6 kinase (RSK), serum response factor (SRF), E26 transformation-specific transcription factors (ETS) and ETS like-1 protein to regulate the transcription and translation of corresponding target genes, thus participating in the regulation of cell proliferation, differentiation, migration and other life activities^44–46^.

#### The PI3K-AKT-mTOR pathway

KRAS was also found to be involved in the PI3K-AKT-mTOR pathway, which is considered to play an important role in cell life activities such as cell proliferation, differentiation, apoptosis and glucose transport and has a great influence on the generation of tumour resistance^47^. Activated KRAS can activate PI3K by binding to its p110 subunit. Activated PI3K-catalysed phosphatidylinositol 4,5-bisphosphate (PIP2) is converted to phosphatidylinositol 3,4,5-trisphosphate (PIP3)^48^. PIP3 promotes phosphoinositide-dependent kinase 1 (PDK1) to phosphorylate AKT at Thr308. mTOR complex 2 further phosphorylates the serine phosphorylation site of AKT (Ser473), resulting in full AKT activation^49^. Activated AKT enters the nucleus, activates or inhibits many downstream pathways, and regulates cell proliferation, apoptosis and metabolic processes^50^. On the one hand, AKT can directly activate mTOR target proteins, which play an important role in cell proliferation, survival, metabolism, protein synthesis, and transcription^51^. On the other hand, AKT phosphorylates and activates Bcl-XL/Bcl-2-associated death promoters (BADs), facilitating the binding of BAD to the companion protein 14-3-3 instead of Bcl-2/Bcl-XL, thus inhibiting apoptosis^52^.

#### Other signalling pathways

**Fig. 2:**
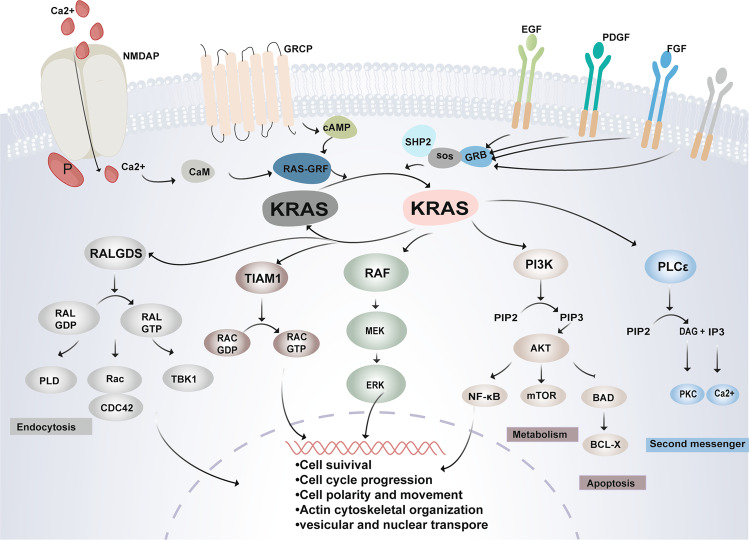
The regulation of KRAS activation and signal transduction. The canonical and well-known pattern of activating KRAS is dependent on correct membrane localisation and adjacent activation of membrane receptors. In the resting state, KRAS normally binds with GDP in an inactivated state. When the extracellular growth factors such as EGF transmit signals to receptors, the SOS, a kind of GEF, interacts with the KRAS-GDP complex leading to the release of GDP and the replacement of GTP. The tether of GTP and KRAS induces structural changes of switch I and switch II, thereby activating KRAS. In contrast, GAPs enhance intrinsic GTPase activity in KRAS to accelerate the reaction in which GTP is hydrolysed to GDP. The KRAS cycle between the activated and inactivated conformations functions as a finely regulated molecular switch that controls multiple signalling cascades, including the canonical RAF-MEK-ERK pathway, which controls proliferation; PI3K-AKT-mTOR pathway, which promotes cell survival; and other signalling pathways, which are required for KRAS-dependent tumour growth and endocytosis, and cytoskeletal organisation

RAL guanine nucleotide dissociation stimulator (RalGDS) is a downstream signalling protein of KRAS that functions as a GTP/GDP exchange factor to promote the GDP/GTP conversion of RAS-like protein (RAL)^53–55^. Downstream effector factors of RAL proteins include Rac/cell division cycle 42 (Cdc42) associated with cell migration, TANK binding kinase 1 (TBK1) associated with viral immunity, and phospholipase D (PLD) associated with endocytosis. KRAS also regulates TIAM1 and RAC1-specific guanine nucleotide exchange factors, to activate RAC1 signalling pathways that affect cell shape, migration, adhesion, actin cytoskeleton formation, endocytosis, and membrane trafficking^56^. In addition, KRAS can also regulate phosphatidylinositol signal pathway by activating PLCε^57^. In short, KRAS-mediated signal networks are complex and related to a variety of life activities (Fig. 2).

## KRAS mutations and cancer

### Mutation characteristics of the oncogene KRAS

**Fig. 3:**
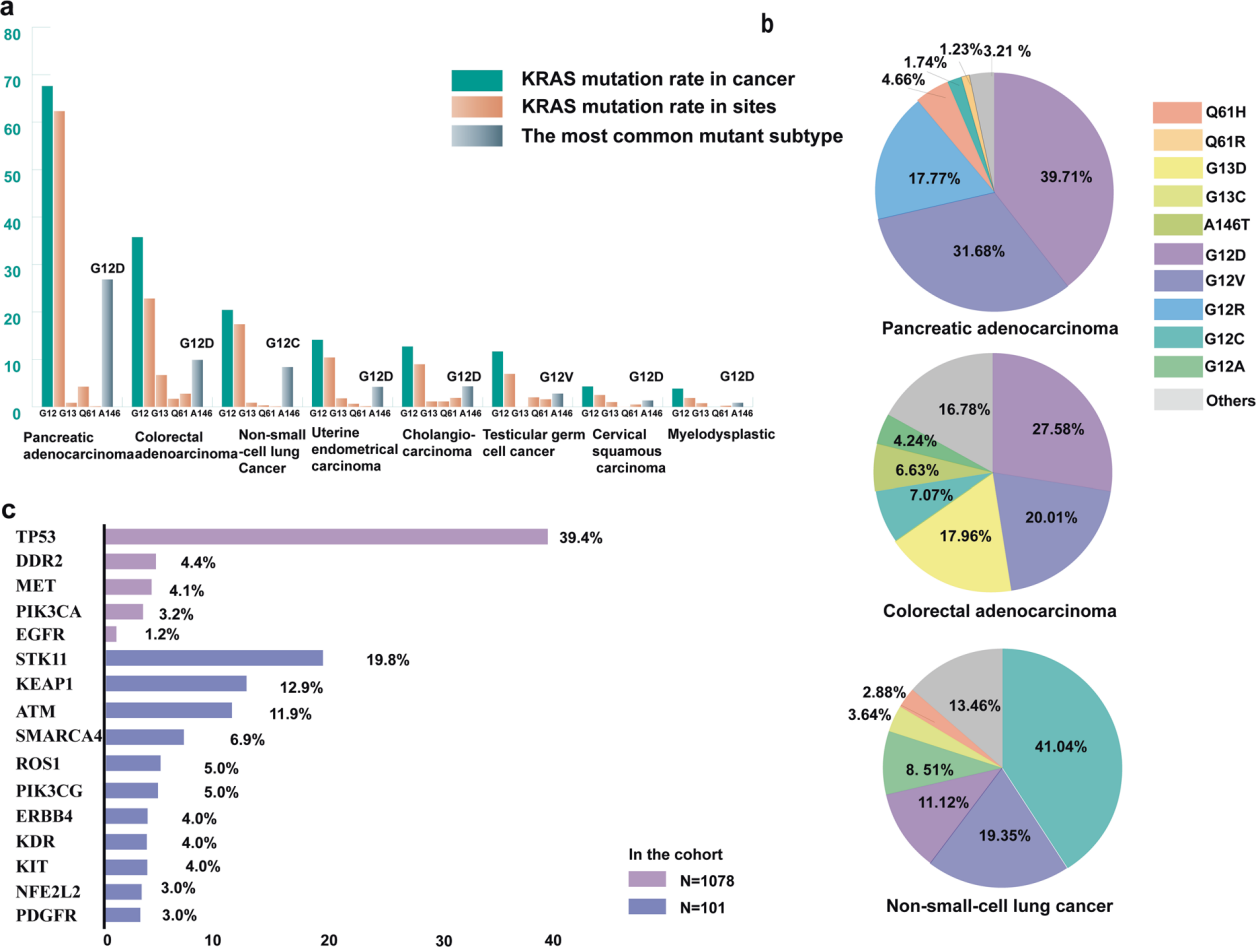
KRAS mutation in cancer. **a** The frequency of KRAS mutations across tumour types, including the mutation frequency of common sites and the subtype with the highest mutation rate in different tumour types. KRAS mutations are characterised by single-base missense mutations, 98% of which are found at codon 12, codon 13, or codon 61. Please refer to Table 1 for specific figures. **b** Specific mutant subtypes and percentages were represented in the top three cancers with the highest mutation rates of KRAS including pancreatic cancer, colorectal cancer, and nonsmall-cell lung cancer. **c** Frequency of co-occurring aberrations in KRAS mutant cells. Only a mutant prevalence of at least 3% is shown in addition to EGFR mutation, given the important effect of EGFR mutation on NSCLC. TP53: tumour protein p53 gene; DDR2: discoidin domain receptor tyrosine kinase 2 gene; MET: MNNG HOST Transforming gene; PIK3CA: phosphatidylinositol-4,5-bisphosphate 3-kinase catalytic subunit alpha gene; STK11: serine/threonine kinase 11 gene; KEAP1: kelch-like ECH-associated protein 1 gene; ATM: ATM serine/threonine kinase gene, PIK3CG: phosphatidylinositol-4,5-bisphosphate 3-kinase catalytic subunit gamma gene; ERBB4: erb-b2 receptor tyrosine kinase 4 gene; KDR: kinase insert domain receptor gene; KIT: KIT proto-oncogene receptor tyrosine kinase gene; NFE2L2 nuclear factor erythroid 2, like 2 gene; PDGFR previous symbol of PDGFRB (platelet-derived growth factor receptor beta gene). Data acquired from The Cancer Genome Atlas (pan-Cancer) from cBioPortal

KRAS is the most commonly mutated member of the RAS family and is considered to be the most common oncogenic gene driver in human cancers^58,59^. KRAS mutations are most common in PDAC, CRC, and NSCLC. The profile of KRAS mutations differs significantly among different cancer types (Table 1). KRAS mutations are dominated by single-base missense mutations, 98% of which are found at codon 12 (G12), codon 13 (G13), or codon 61 (Q61)^60^. Notably, KRAS mutations occur in many cancers with different mutation frequencies, but there is also a large variation in mutation subtypes (Fig. 3a, b). For example, in NSCLC, KRAS mutations account for 20.4% of KRAS, and the dominant substitution is G12C (glycine (GGT) to cysteine (TGT)), while KRAS mutation accounts for up to 67.6% of KRAS in pancreatic adenocarcinoma, and KRAS (G12D) is the dominant mutant subtype. It is important to note that the mutation rate of KRAS in the pancreas was 67.6% based on this acquired data analysis, which is lower than the commonly cited 90%. The incidence of KRAS mutations between 25 and 35% in smokers and 5% in nonsmokers has been reported, and smoking is usually considered to be a relevant factor^61^. Furthermore, the profiles of KRAS mutations are distinct in smokers and nonsmokers, and not all mutations in KRAS are driver mutations. For instance, KRAS (G12C) is usually found in heavily smoking patients, while KRAS (G12D) is more usually identified in tumours from nonsmoking patients^62^.

**Table 1:** The frequency of KRAS mutations in cancer

		KRAS mutations (%)							
Tumour types	Sample	Total rate	Mutation sites					Top 1	
			G12	G13	Q61	A146	Other		
Pancreatic adenocarcinoma	1207	67.61	62.30	0.83	4.23	0.08	0.17	G12D	26.84
Colorectal adenocarcinoma	3953	35.77	22.82	6.68	1.67	2.76	1.85	G12D	9.87
Nonsmall-cell lung cancer	7135	20.42	17.39	0.85	0.31	0.06	1.81	G12C	8.38
Cholangiocarcinoma	1072	12.69	8.96	1.12	1.12	1.87	0.37	G12D	4.29
Uterine endometrial carcinoma	1907	14.11	10.38	1.78	0.63	0.10	1.21	G12D	4.20
Testicular germ cell cancer	506	11.66	6.92	0.00	1.98	1.58	1.19	G12V	2.77
Cervical squamous cell carcinoma	607	4.28	2.47	0.99	0.00	0.49	0.33	G12D	1.32
Myelodysplastic	6940	3.83	1.86	0.75	0.29	0.23	0.71	G12D	0.84

Data sources from cBioPortal.org. G12: codon 12 encoding glycine; G13: codon 13 encoding glycine; Q61: codon 61 encoding glutamine; A146: codon 146 encoding

### Biochemical heterogeneity of KRAS mutations

The conventional understanding of KRAS mutations is that alterations in KRAS proteins caused by mutations impede the interaction of KRAS with GAPs and the hydrolysis of GTP bound to KRAS, leaving KRAS in a constitutively active state^63,64^. Numerous studies have shown biochemical heterogeneity of KRAS mutations in various aspects, including intrinsic GTPase activity and the affinity of effectors and metastatic sites. For instance, alterations in codons 12, 13, and 61 usually lead to impaired intrinsic GTPase activity of KRAS, distinct from KRAS (G12D), KRAS (G12C), and KRAS (G13D)^65^. The type of KRAS mutation can also affect the interaction with its effectors. Moreover, KRAS (G12) and KRAS (Q61) are insensitive to neurofibromin 1-mediated hydrolysis, whereas KRAS (G13) is partially sensitive to neurofibromin 1, a kind of GAP^66^. In addition, cells containing KRAS (G12C) or KRAS (G12V) have increased levels of RAS-related protein (RAL) A/B signalling and decreased levels of phosphorylation of protein kinase B (AKT) compared with cells with other KRAS mutations or wild-type cells^67^. However, cell lines with KRAS (G12D) have higher levels of phosphorylated AKT^68^. According to the binding affinity of KRAS with RAF, an important effector, the mutation can be divided into two classes: high affinity (G12A, G12V, G12R, Q61H, and Q61L) and low affinity (G12R, G12D, and G12V). Different KRAS-mutant subtypes have different sensitivities to targeted therapy. For example, a study performing high-fidelity CRISPR-based engineering found that KRAS (G12D) is sensitive to EGFR inhibition in pancreatic cancer models, while KRAS (G12C) mutants selectively respond only to covalent G12C inhibitors when EGFR is inhibited^69^. In terms of metastasis, patients with KRAS mutations have a greater opportunity for lung or brain metastases^70^. Another study revealed that patients harbouring G12V mutations often have pleuropericardial metastases, while patients harbouring G12C and G12D mutations usually have bone metastases^71,72^. Notably, not all KRAS-mutant tumours are KRAS-dependent tumours. More relevant future studies are needed to expand the heterogeneity map of KRAS mutations and provide a theoretical basis for more accurate individualised treatments.

### Comutations of KRAS mutations

In addition to the different mutation levels and mutation subtypes in different cancer tissues, KRAS mutations may have different comutations, which may influence the function of KRAS and the occurrence and development of tumours. Analysing 1078 NSCLCs harbouring KRAS mutations, 557 patients (53.5%) harboured comutations, and of 14 analysed genes, tumour protein p53 gene (TP53) mutation was the most common comutation, accounting for 39. 4%^73^. In addition, 101 patients in the above cohort were analysed for an additional 14 genes, such as the serine/threonine kinase 11 gene (STK11) and kelch-like ECH-associated protein 1 gene (Fig. 1c)^73^. Interestingly, the study indicated that EGFR mutations were detected in KRAS-mutated NSCLC, albeit with a very low frequency of less than 1%. This is controversial because it is widely believed that EGFR and KRAS mutations are mutually exclusive^74,75^. In the future, larger amounts of clinical data will be needed to draw more comprehensive and rational conclusions. There were distinct biological behaviours in different subgroups, altering outcomes in patients^76^. For example, tumours with KRAS/STK11 comutation commonly have a tumour microenvironment (TME) with poor immune response, lacking CD8 + tumour-infiltrating lymphocytes but including an abundance of T regulator cells. However, CD8 + tumour-infiltrating lymphocytes and activated dendritic cells are rich, and there are few T regulator cells in the TMEt with the KRAS/P53 comutation^77^. In addition, clinical evidence has shown that patients harbouring ALK or EGFR/KRAS commutations respond poorly to tyrosine kinase inhibitor therapy^78^. In 11,951 Chinese tumour samples, KRAS mutations accounted for 16.6%, of which KRAS (G12C) accounted for 14.5%. Almost all patients (99.6%) with G12C mutations were associated with genomic aberrations that were associated with the RAS/RTK pathway^79^. In general, when treating KRAS-driven tumours, it is necessary to pay attention to other gene changes and pay attention to individualised treatment (Fig. 3c).

### KRAS mutations and TME

There is growing evidence that patterns of genetic changes influence the immune environment of cancer. Increasing evidence shows that KRAS mutations in tumours not only dedicate the intrinsic characteristics of tumours, such as survival and proliferation but also form a tumour microenvironment (TME), especially affecting immune cells in the TME and eventually resulting in tumour progression and immune escape^80,81^.

### KRAS mutations and inflammatory TME

The TME in the presence of KRAS mutations often appears to be inflammatory and infiltrated with multiple immune cells. This inflammatory microenvironment is facilitated by high levels of a series of inflammatory cytokine and chemokine factors mediated by the KRAS signalling pathway^81–83^. The overactivation of KRAS signalling has been shown to enhance the secretion of interleukin-6 (IL-6), which is necessary for tumour initiation and progression and is important for the crosstalk between tumours and inflammation^84,85^. KRAS-induced IL-6 promoted the activation of Janus activated kinase 1 (JAK1) and phosphorylation of signal transducer and activator of transcription 3 (STAT3), contributing to tumorigenic cellular processes in a variety of tumour types^86–88^. In pancreatic cancer, IL-6 not only promotes the occurrence and progression of tumours through JAK1/STAT3 but also activates reactive oxygen species through the ERK pathway^89^. Studies have also shown that IL-6 secreted by myeloid cells can promote pancreatic intraepithelial neoplasia progression and PDAC with KRAS mutations^90^. IL-8 is also related to tumours and inflammation as a C-X-C motif chemokine receptor 2(CXCR2) ligand. IL-8 has been identified as a transcriptional target for KRAS-mediated ERK or PI3K signal transduction, affecting endothelial cell recruitment, tumour-associated inflammation formation, and tumour angiogenesis^90,91^. The role of inflammation in promoting lung cancer has been shown to be mediated in part by activation of the IL-8/CXCR2 pathway and subsequent neutrophil recruitment and release of neutrophil elastase^91^. KRAS/CXCR2 signalling in PDAC has been shown to induce the generation of cancer-associated fibroblasts with enhanced secretory function that mainly secrete protumorigenic cytokines^92^. In addition, KRAS has been reported to be involved in the formation of PDAC and the development of NSCLC by promoting the production of IL-1α and the activation of IKKβ/NF-κB^93,94^.

Several chemokines are involved in the proinflammatory TME. Although elevated levels of C-C chemokine ligand 5(CCL5) were detected in a variety of tumours and were associated with tumour progression, CCL5 also exhibited antitumour abilities by recruiting T cells and dendritic cells to the TME. In KRAS-driven lung cancer, the IKK-related kinases TBK1 and IKKε downstream of KRAS signalling increased CCL5 levels^95^. KRAS mutations in pancreatic acinar cells were able to upregulate intercellular adhesion molecule 1, resulting in chemical absorption by M1 macrophages^96^. M1 macrophages release stromal degrading enzymes, such as matrix metalloproteinase-9 and cytokines such as tumour necrosis factor (TNF), which contribute to the formation of an inflammatory environment. Several KRAS-mutant TMEs showed increased Th17 cells and significantly increased IL-17 levels. For example, in pancreatic KRAS mouse genetic models, increased numbers of Th17 cells were observed in PDAC and participated in tumour initiation and progression by the production of IL-17^97^. Recently, KRAS-mediated activation of NLRP3, which forms inflammasomes, was shown to affect the pathogenesis of KRAS-driven myeloproliferation^98^. In conclusion, this evidence supports that KRAS induces an inflammatory TME and facilitates the occurrence and development of tumours by inducing inflammation.

### KRAS mutations and tumour immune escape

**Fig. 4:**
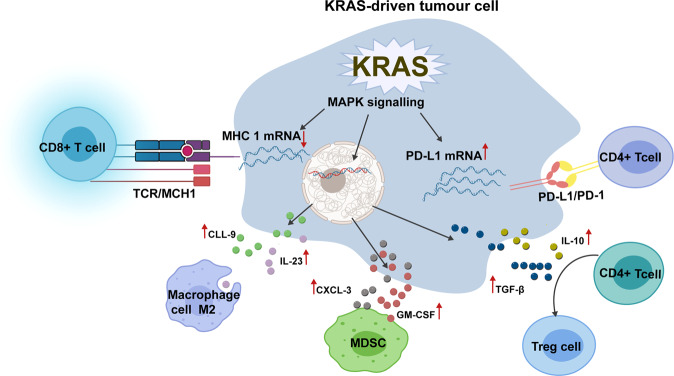
KRAS-mediated immune escape in tumour microenvironment. KRAS mediates immune escape in the tumour macroenvironment by upregulating PD-L1expression, downregulating MHC1expression of tumour cells, and enhancing the secretion of a variety of cytokines and chemokines to recruit immunosuppressive immune cells. The black arrow represents facilitation, and the opposite red arrow represents inhibition. MDSCs: myeloid-derived suppressor cells; Treg cells: regulatory T cells; IL-10: interleukin-10; TGF-β: transforming growth factor-β; GM-CSF: granulocyte-macrophage colony-stimulating factor; IL-23: interleukin-23

The TME of most solid tumours is infiltrated by multiple immune cells, including T cells and macrophages. However, many of these immune cells have been cultured in the TME and ultimately possess immunosuppressive properties, such as regulatory T cells (Tregs), myeloid-derived suppressor cells (MDSCs), tumour-associated macrophages (TAMs), neutrophils, and mast cells. KRAS-mediated signalling plays an important role in the formation of an immunosuppressive TME and the modification of immune cells (Fig. 4)^99^.

KRAS mutations mediate immune escape by regulating the intrinsic characteristics of tumour cells. In KRAS-driven tumours, mutant KRAS mediates tumour immune escape by upregulating PD-L1 expression. There is growing evidence that KRAS (G12C), KRAS (G12V), KRAS (G12D), and KRAS (G13D) mutations are associated with high PD-L1 expression in lung cancer^100,101^. The mutant KRAS signalling pathway upregulates the expression of PD-L1 in tumour cells by improving the stability of PD-L1 mRNA^102^. Although the AU-rich binding protein tristerrolin (TTP) reduced the expression of PD-L1 through AU-rich elements in the PD-L1 mRNA 3’UTR, the MEK signal downstream of KRAS phosphorylates and inhibits TTP, thus increasing the expression level of PD-L1^102^. Another study reported another mechanism of KRAS (G12V)-mediated upregulation of PD-L1 expression by promoting ROS production and inducing FGFR1 expression^103^. After antioxidant treatment, PD-L1 expression in KRAS-mutant cells was largely eliminated, and FGFR1 gene knockout also led to reduced PD-L1 expression and impaired tumour growth in vivo^103^. Notably, the PD-L1 expression levels in CRC patients were not significantly associated with KRAS mutations, unlike LAC. In addition to affecting PD-L1 expression, KRAS mutation impacted the immunogenicity of tumour cells by downregulating the expression of major histocompatibility complex (MHC) class I molecules in a CRC cell line with the KRAS (G13D) mutation^104^. MHC1 is essential for antigen presentation and antigen recognition by T cells. Downregulation of MHC 1 severely attenuated the tumour killing effect of T cells, especially CD8 + cytotoxic T cells. The knockdown of mutant KRAS (G12D) in a poorly immunogenic CRC model improved the immune response and caused tumour regression^105^.

In addition to affecting tumour cells, KRAS mutations play an immune evasion role by affecting immune cells in the TME, such as the acquisition and recruitment of inhibitory phenotypes of immune cells. KRAS mutations induce CD4^+^ T cells in the TME to transform into immunosuppressive Treg cells by promoting the secretion of related cytokines. The KRAS (G12C)-mediated phenotypic transformation of T cells has been shown to be a consequence of the secretion of IL-10 and TGF-β1 mediated by MEK/ERK/AP-1 signalling in CRC^106^. In the KRAS transgenic lung cancer model, the gene ablation of Treg cells resulted in inhibition of the occurrence and progression of lung cancer, indicating the necessity of Treg cells in the development of lung tumours^107^. In addition, the KRAS (G12V) and KRAS (G12D) mutations enhanced the infiltration of MDSCs in the TME by upregulating GM-CSF in PDAC and CRC, thereby leading to antitumour immune escape^108,109^. Another study has shown that KRAS (G12D) appears to inhibit the secretion of interferon regulatory factor 2 (IRF2), thereby promoting increased CXCL3 secretion, which acts on CXCR2 on MDSCs, leading to MDSC migration to the TME^110^.

In KRAS-mutated tumours, other oncogenes or tumour suppressor genes may also be abnormal and may participate in immune escape together with KRAS mutation. For example, in lung cancer, coactivation of KRAS (G12D) and MYC drove the aggregation of anti-inflammatory macrophages, but the absence of T, B, and NK cells was due to the effect of CCL9 and IL-23^111^. In PDAC, studies have shown that KRAS (G12D) and TP53 jointly activate the ARF6/AMAP1 pathway, affect the level and presentation of PD-L1, and promote tumour development and immune invasion^112^. Through the analysis of clinical data of LAC patients, the expression of PD-L1 in tumours in the TP53/KRAS comutation group was increased, and the proportion of CD8 + T cells in TME was higher, which was consistent with the clinical benefit of TP53, KRAS or TP53/KRAS-mutated cancer patients after treatment with PD-1 inhibitors^113^. In addition, patients with STK11/LKB1 mutations were resistant to PD-1 inhibitors in KRAS-mutant LAC, suggesting a new mechanism of resistance^103^. In general, KRAS comutations affect the immune regulation of the KRAS-driven TME by recruiting immunosuppressive cells and increasing PD-L1 expression.

### Successfully targeting KRAS (G12C)

KRAS plays a central role in signal transduction, and KRAS mutations are closely related to tumour initiation and development. Successful targeting of mutant KRAS will lead to a new platform for targeted oncology therapy. However, after nearly 40 years of effort, KRAS remains an unsolved puzzle. Due to the difficulty of direct targeting, researchers switched to other important molecules in the KRAS signalling pathway, such as RAF, ERK, and MEK. However, there has been no significant success in KRAS-driven tumours. KRAS-mutant tumours have obvious heterogeneity, which partly explains the poor efficacy of nonspecifically targeting KRAS. Selective inhibitors targeting specific KRAS mutations are urgently needed to effectively inhibit different mutant KRAS functions in line with the requirements of precision oncology.

**Fig. 5:**
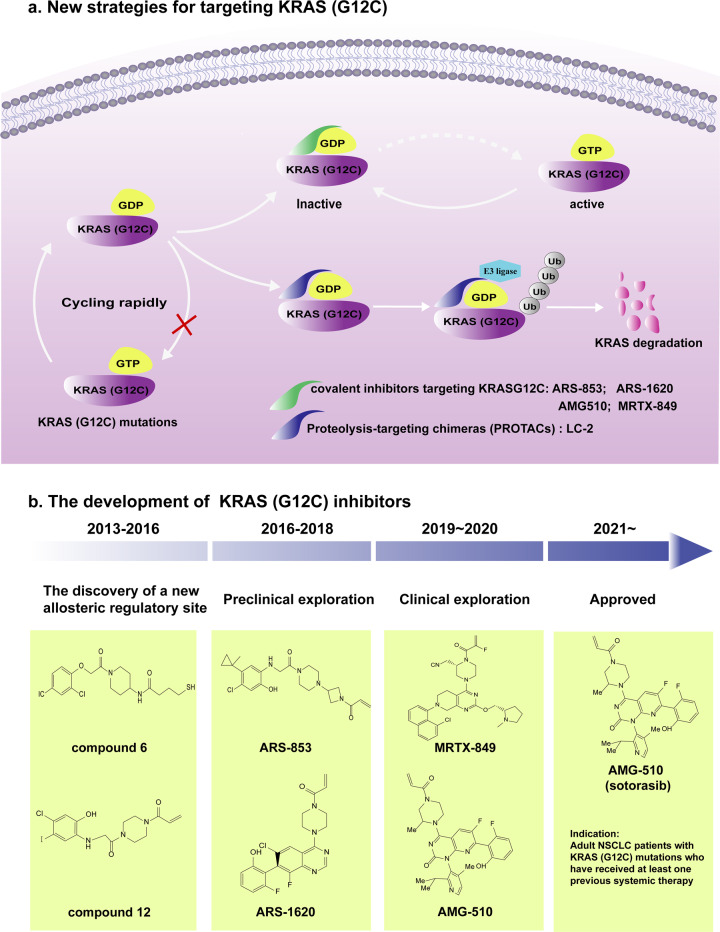
Current targeted strategies for KRAS (G12C). **a** Despite the mutation occurring in KRAS, KRAS (G12C) still continues to perform the KRAS-GDP/GTP cycle. Covalent inhibitors such as AMG510 and MRTX849 lock KRAS (G12C) in inactivated GDP-bound state, thus decreasing functional KRAS. Another strategy is to increase the degradation of mutant KRAS (G12C) proteins. Based on the previously described KRAS (G12C) covalent inhibitors, LC-2, an endogenous KRAS (G12C) degrader, has been developed to promote KRAS (G12C) degradation. **b** The development and chemical structures of KRAS (G12C) inhibitors

The first signs of the dawn appear on the horizon for one specific mutation, KRAS (G12C). Unlike KRAS (G12D) and KRAS (G12V), KRAS (G12C) can maintain alternative interactions with its downstream effectors through an active cycle between the GDP-bound and GTP-bound states^114^. This difference enables KRAS (G12C) to be locked into an inactive conformation by reacting with cysteine residues. For instance, SML-8-73-1, a GDP analogue in which beta phosphate binds an electrophilic chloroacetamide, can covalently react with cysteine 12 (Cys12) of KRAS (G12C). However, SML-8-73-1 appears to be cell impermeable due to possessing two negative charges^115^. SML-10-70-1, a caged version of SML-8-73-1, can penetrate cells but lacks selectivity and antitumour activity^116^. Fortunately, another attractive target has been identified: the thiol group in the cysteine residue forms a disulphide bridge with specific inhibitors with prolonged target engagement. Notably, this strategy exhibits not only remarkable selectivity but also greater activity because Cys12 is located near switch regions involved in the interaction with effectors and the nucleotide. Novel inhibitors targeting KRAS (G12C) have displayed promising results in preclinical and clinical trials, showing promise for targeting KRAS, which was considered to be undruggable in the past (Fig. 5).

### The discovery of a new allosteric regulatory site in KRAS (G12C)

Initially, Shokat and colleagues designed a sequence of small molecule compounds based on the specific nucleophilic properties of the mutant half cystine thiols for targeting KRAS (G12C)^117^. The crystal structure of compound 6 combined with the KRAS (G12C)-GDP complex clearly indicates that compound six extends from Cys12 into an adjacent pocket named the switch-II pocket (S-IIP), rather than binding to the nucleotide pocket^117^. Notably, S-IIP is not obvious in other published structures of other KRAS or isoforms, although a groove is visible in some cases^118,119^. Shokat and colleagues changed the drug-design strategy to focus on acrylamides, vinyl sulfonamides and carbon-based electrophiles rather than continuing with disulfide-based compounds. This strategy was not only chemically selective but irreversible. Finally, they obtained the most potent acrylamide 12. Cells containing the KRAS (G12C) mutation exhibited reduced viability and increased cell apoptosis when treated with compound 12 compared with cells without the KRAS (G12C) mutation^117^. Overall, a new allosteric pocket in KRAS (G12C) and relatively specific inhibitors were identified, providing structure-based validation that KRAS is targetable^58^.

### Preclinical exploration of KRAS (G12C) inhibitors

The discovery of a new allosteric site has reignited researchers’ enthusiasm for targeting KRAS (G12C). Based on previous compounds reported by Shokat and colleagues, a series of compounds were constantly developed to seek the path to success, including ARS-853 and ARS-1620 (Fig. 4b). ARS-853 was developed from first-generation compounds 6 and 12 by optimising electrophilic localisation and modifying the scaffold that interacts with a hydrophobic portion of the S-IIP^120,121^. Studies have shown that ARS-853 inhibits the KRAS (G12C) protein by locking it in the inactive state of the GDP-GTP binding state cycle. Increasing evidence suggests that KRAS mutations are unequal and that different KRAS mutations reflect biological heterogeneity^122^. Furthermore, by using ARS-853 as a molecular tool, a study found that KRAS (G12C) is rapidly cycling rather than in a statically active state, that KRAS (G12C)-mediated signalling can be regulated by upstream effectors, and that cell viability and growth depend on KRAS mutations to varying degrees even within KRAS^G12^ cancer cell lines^120,123^.

ARS-1620 is the first proof-of-concept molecule to demonstrate the feasibility of targeting KRAS (G12C) in vivo. According to the cocrystal structure, ARS-1620 possesses an additional covalent interaction with His95 of KRAS (G12C), which provides a more stable and preferred conformation than ARS-853^124^. In brief, ARS-1620 is an orally selective and potent agent in a variety of models. An apparent paradox is why a target without conventional binding pockets can nevertheless be inhibited by small molecules with powerful efficiency from the nanomolar to the low micromolar range. The study of Rasmus Hansen and colleagues demonstrated that inhibitors exhibit an only weak and reversible binding affinity for KRAS (G12C), but the chemical reaction between the targeted cysteine and inhibitors is powerful and is accelerated by KRAS (G12C) as an enzyme that specifically catalyses covalent bond formation between groups with low intrinsic reactivity^125^. Therefore, the main driver of potency is KRAS-mediated acceleration of the chemical reactions of specific KRAS (G12C) inhibitors.

### Clinical exploration of KRAS (G12C) inhibitors

#### AMG510 (sotorasib)

AMG510 is the first small molecule inhibitor specifically targeting KRAS (G12C) to enter clinical trials (NCT03600883), which specifically and irreversibly bind to Cys12 in the inducible S-IIP and lock KRAS (G12C) protein in an inactive status^126,127^. AMG510 is additionally combined with a novel surface groove formed by an alternative orientation of His95 on KRAS, resulting in a tenfold improvement in efficiency compared to ARS-1620^128,129^. Excitingly, AMG510 was approved by the U.S. Food and Drug Administration, on May 28, 2021, as the first treatment for adult patients with NSCLC whose tumours harbour KRAS (G12C) mutations and who have received at least one prior systemic therapy^130^. This is a milestone because it is the first drug to directly target mutated KRAS. The full Phase I cohort (*n* = 129) receiving daily AMG510 monotherapy in the clinical trial (NCT03600883) was completed^131^. This monotherapy trial showed responses across all dose levels tested, and no dose-limiting toxicity or treatment-related deaths were observed^131^. A total of 56.6% patients (*n* = 76) reported treatment-related adverse events, of which 11.6% of patients (*n* = 15) reported grade 3 or 4 events, including reversible elevations in alanine aminotransferase levels, diarrhoea, vomiting, and anaemia. In the subgroup with NSCLC (*n* = 59), the ORR and DCR were 32.2% (*n* = 19) and 88.1%, respectively. Simultaneously, a median progression-free survival of 6.3 months and the recommended phase II dose of 960 mg daily were identified^131^. On January 28, 2021, Amgen revealed the results of a phase II clinical trial (NCT03600883) evaluating 126 patients with KRAS (G12C)-mutant NSCLC. Surprisingly, an ORR of 37.1%, including 3 complete responses and 43 partial responses, and a DCR of 80.6% were reported. A median duration of response of 10 months and a median progression-free survival of 6.8 months were also confirmed, in line with earlier Phase I results. The security of AMG510 was further confirmed. Grade 3 adverse events were reported in 19.8% of patients, and grade 4 adverse events were reported in only 1 patient (0.8%), with no treatment-related deaths. These attractive results make AMG510 the first approved drug to treat KRAS (G12C)-mutant NSCLC. There are other ongoing clinical trials to further assess AMG510, such as NCT04185883, which will assess the safety and feasibility of various combinational therapies; NCT04303780, which compares AMG510 with docetaxel; and NCT04625647, which will evaluate the response rate of AMG510 in participants with KRAS (G12C)-mutated stage IV or recurrent nonsquamous NSCLC (Table 2).

**Table 2:** The clinical development of KRAS (G12C) inhibitors

ClinicalTrials.gov registration	Drug	Disease setting	Study phase	Recruitment status
NCT03600883	AMG510	Solid tumours	I/II	Recruiting
NCT04667234	AMG510	Metastatic NSCLC	I	Recruiting
NCT04380753	AMG510	Metastatic solid tumours	I	Recruiting
NCT04625647	AMG510	Nonsquamous NSCLC	II	Recruiting
NCT03785249	MRTX849	CRC; NSCLC	I/II	Recruiting
NCT04165031	ARS-3248	CRC; NSCLC	I	Completed
NCT04165031	LY3499446	Solid tumours	I/II	Terminated
NCT04585035	D-1553	Solid tumours	I/II	Recruiting
NCT04699188	JDQ443	Solid tumours	I/II	Recruiting
NCT04449874	GDC-6036	Advanced Solid Tumours	I a/I b	Recruiting

NSCLC: nonsmall-cell lung cancer; CRC: Colorectal adenocarcinoma. Data from ClinicalTrial.gov, accessed September 18, 2021

#### MRTX849 (adagrasib)

The ongoing clinical study of MRTX849 also offers hope for the successful targeting of KRAS (G12C) proteins^132^. The preliminary results of a multiple expansion study evaluating MRTX849 (NCT03785249) were reported at the 2019 EORTC-NCI-AACR annual symposium. In the cohort of 12 patients, including six NSCLC patients, most patients received a 600 mg dose twice daily, of whom three NSCLC patients reported unconfirmed partial responses and other patients experienced stable disease. MRTX849 was well tolerated in most patients with only grade 1 adverse events, of which diarrhoea and nausea were the most common. Another phase I/II study (NCT04330664) is also ongoing to assess MRTX849 in combination with NO155, an SHP2 inhibitor, in patients with the KRAS (G12C) mutation (Table 3).

**Table 3:** Combination therapy of KRAS (G12C) inhibitors in clinical trials

ClinicalTrials.gov registration	Drug	Disease setting	Study phase	Recruitment status
Combined with chemotherapy				
NCT04303780	AMG510 Docetaxel	NSCLC	III	Recruiting
NCT04185883	AMG510 Docetaxel	Advanced NSCLC	I b	Recruiting
NCT04165031	LY3499446 Docetaxel	Solid tumours	I/II	Terminated
NCT04685135	MRTX849 Docetaxel	Metastatic NSCLC	III	Recruiting
Combined with targeted therapy				
NCT04185883	AMG510 Erlotinib	Solid tumours	I b	Recruiting
NCT04185883	AMG510 TNO155	Solid tumours	I b	Recruiting
NCT04185883	AMG510 Selumetinib	Solid tumours	I b	Recruiting
NCT04185883	AMG510 Everolimus	Solid tumours	I b	Recruiting
NCT03785249	MRTX849 Afatinib	NSCLC	I/II	Recruiting
NCT03785249	MRTX849 Cetuximab	Solid tumours	I/II	Recruiting
NCT04793958	MRTX849 Cetuximab	Metastatic CRC	III	Recruiting
NCT04330664	MRTX849 TNO155	NSCLC	I/II	Recruiting
NCT04165031	LY3499446 Abemaciclib	Advanced NSCLC	I/II	Terminated
NCT04165031	LY3499446 Erlotinib	Advanced NSCLC	I/II	Active not recruiting
NCT04449874	GDC-6036 Erlotinib	Solid tumours	I a/I b	Recruiting
NCT04449874	GDC-6036 Cetuximab	Solid tumours	I a/I b	Recruiting
NCT04449874	GDC-6036 Bevacizumab	Solid tumours	I a/I b	Recruiting
Combined with immune therapy				
NCT03600883	AMG510 Pembrolizumab	NSCLC	II	Recruiting
NCT03600883	AMG510 Atezolizumab	NSCLC	II	Recruiting
NCT03785249	MRTX849 Pembrolizumab	NSCLC	I/II	Recruiting
NCT04613596	MRTX849 Pembrolizumab	NSCLC	II	Not yet recruiting
NCT04449874	GDC-6036 Atezolizumab	Solid tumours	I a/I b	Recruiting

NSCLC: nonsmall-cell lung cancer; CRC: colorectal adenocarcinoma. Data from ClinicalTrial.gov, accessed September 18, 2021

Other specific KRAS (G12C) inhibitors from other companies are also under investigation in clinical trials, including LY3499446, D-1553, and ARS-3248/JNJ-74699157, which are next-generation ARS-1620 inhibitors (NCT04006301, NCT04165031, and NCT04585035) (Table 2). Notably, the first KRAS (G12C) inhibitor, LY3499446, from Eli Lilly, has been discontinued due to safety concerns, but at the 2021 AACR meeting, they presented preclinical results for another inhibitor, LY3537982. In KRAS (G12C) mutant H358 cells, the inhibitory activity of LY3537982 was at least 10 times higher than that of AMG510 and MRTX849. We look forward to the further results of LY3537982 in preclinical and clinical trials.

### Proteolysis-targeting chimaeras (PROTACs) targeting KRAS (G12C) proteins

Compared with direct inhibition, degradation could be a more potent strategy that affects cell proliferation and downstream signalling responses^133,134^. Based on covalent inhibitors targeting KRAS (G12C), PROTACs, as small molecule degraders, are designed to target KRAS (G12C). The E3 ligase is able to ubiquitinate proximal lysine residues of KRAS (G12C) by PROTACs that simultaneously engage KRAS (G12C) and E3 ligase substrate receptor proteins, such as the cerebellum (CRBN). A covalently degrading molecule based on a thalidomide scaffold and ARS-1620 has been reported to degrade the artificial GFP-KRAS (G12C) fusion protein in reporter cells^135^. Subsequently, LC-2, the first endogenous KRAS (G12C) degrader, was reported to rapidly engage and continuously degrade KRAS (G12C), which consisted of MRTX849 and the VHL E3 ligase ligand^136^. LC-2-mediated KRAS (G12C) degradation requires an intact proteasome system and VHL E3 ligase complex assembly, of which neddylation is important for the formation and function of the VHL E3 ligase complex; nevertheless, the lysosomal pathway is nonessential^137^. After treatment with LC-2, multiple cancer cells demonstrated ERK phosphorylation inhibition^136^. Moreover, there has been an unmet need for reversible degraders attacking KRAS (G12C) because LC-2 cannot be involved in multiple catalytic cycles of degradation, limiting its potency due to its covalent nature^138^. The development of PROPAC_S_ is considered to be an important direction for antitumour therapy and has broad prospects, although in vivo data have yet to be obtained (Fig. 5a).

## Targeting other KRAS mutations and indirect strategies

### Targeting other KRAS mutations

The successful development of KRAS (G12C) inhibitors is dependent on the covalent inhibition of cysteine and high GTPase activity of KRAS (G12C), which is not fully present in other KRAS-mutant types^114^. New strategies are therefore needed to target other common KRAS mutations, such as KRAS (G12D) and KRAS (G12V). By screening a random peptide library for purified recombinant KRAS (G12D) exhibited on the T7 phage, researchers obtained a novel and selective inhibitory peptide (KRpep-2d) to KRAS (G12D), as in the first report of a KRAS (G12D)-selective inhibitor^139^. Then, KS-58 based on KRpep-2d was reported as the first KRAS (G12D)-selective inhibitory peptide presenting in vivo anticancer activity^140^. Another study suggested that CRISPR/Cas9 could be delivered via exosomes to target KRAS (G12D) in models of pancreatic cancer^141^. Koide and his colleagues successfully developed a noncovalent inhibitor, termed 12VC1, that can highly selectively bind to the active states of KRAS (G12V) and KRAS (G12C) in vivo and in vitro^142^. Based on this monomer, PROTAC-like fusion proteins were developed, which could electively and effectively increase the degradation of KRAS (G12V). Using a structure-based drug design, Kessler and colleagues discovered that Bi-2852, a nonspecific KRAS inhibitor, binds with nanomolar affinity to a pocket between switches I and II on active and inactive KRAS in vitro^143^. Overall, the development of specific inhibitors for other KRAS mutations is still in its infancy and is a long way from reaching clinical trials. However, the emergence of new approaches also offers hope for successfully targeting different KRAS-mutated subtypes.

### Indirect strategies of targeting KRAS

For more than 30 years after its discovery, KRAS was considered undruggable target due to the intrinsic characteristics of KRAS proteins. The KRAS is small and has a considerably smooth and shallow surface, resulting in difficulty of small molecule binding to the KRAS. There is no other pocket on the surface of KRAS that can bind to small molecules except the GTP binding pocket, but targeting the GTP binding pocket is quite difficult^144^. Because there is a quite high concentration of GTP in cells under physiological conditions and the affinity between the GTP and KRAS reaches the picomolar level, GTP almost monopolises the sole pocket on the surface of KRAS^145^. These inherent conditions make it nearly impossible to develop competitive KRAS inhibitors. Therefore, various explorations for indirectly targeting KRAS have been performed over the past 40 years, of which most strategies are nonspecific and inefficient, including reducing the expression of KRAS, interrupting the membrane location of KRAS, interfering with the interaction between the KRAS and its effectors, inhibiting upstream and downstream signalling, and synthetic lethality approaches such as cyclin-dependent kinase inhibitors. However, most of these strategies have failed due to a lack of activity or selectivity.

### Reducing the expression of KRAS

RNA-based approaches to degrade overexpressed mRNAs have been shown to be effective and feasible in vivo, as exosomes can act as effective vectors for short interfering RNAs to mediate entry into tumour cells^146,147^. Among them, progress has been made in the development of antisense oligonucleotides (ASOs) targeting KRAS in lung cancer. AZD4785, developed by Macleod and colleagues, is an ASO targeting KRAS with a novel 2’-4’-constrained ethyl modification^148^. KRAS expression was significantly reduced in subcutaneous tumours after treatment with AZD4785, but the reduction was not obvious in clinical trials (NCT03101839).

### Interrupting membranal location of KRAS

Only membrane-bound KRAS can be activated and activate downstream signalling pathways. Therefore, preventing membrane localisation after KRAS translation should be an effective strategy. Farnesyltransferase was first identified as a potential therapeutic target at an early stage due to its key role in the localisation of KRAS^149^. Of several powerful farnesyltransferase inhibitors (FTIs), tiffany and lonafarnib have entered phase III clinical trials^150^. However, the treatment of KRAS-driven cancers ultimately failed due to the lack of antitumour effects^151^. Subsequent findings indictaed that KRAS was successfully localised by modification of prenylation by geranylgeranyl transferase type 1-mediated substitution pathways in the absence of farnesyltransferase^152^. However, cotargeting of farnesyltransferase and geranyl transferase also did not produce significant antitumour effects^153^. Salirasib, as a second-generation FTI, was developed but terminated in phase II trials due to lack of efficacy^154^. Other post-translational modification enzymes, such as RAS-converting enzyme 1 or isoprenyl carboxyl methyltransferase may also be potential therapeutic targets to block KRAS membrane localisation, especially phosphodiesterase-δ, which assists the transport of KRAS from the Golgi apparatus to the plasma membrane^155^. However, relevant studies are still in the preclinical stage and different studies have presented puzzling and different results that need to be further investigated in the future^156,157^.

### Inhibiting KRAS-mediated signal transmission

Protein-protein interactions are an important form of signalling. Therefore, interference in the interaction between the KRAS and other proteins may be a potential therapeutic strategy to block KRAS-mediated signal transmission. GEFs are a class of key proteins of KRAS interaction, such as SOS1. SOS1 inhibitors have also been designed and synthesised to block KRAS-SOS1 interaction^158^. Of them, BI1701963, an orally bioavailable pan-KRAS inhibitor, has entered a phase I clinical trial (NCT04111458), which has an obvious effect in inducing tumour senescence. In addition, stapled peptides, which can block the interaction of KRAS with SOS1 based on a stabilised α-helical structure, are also considered promising as an effective strategy to interfere with the interaction between KRAS and SOS1^159^. In addition, small molecules that directly block the interaction of KRAS and downstream effectors, primarily RAF kinases, have not yet been identified, because the interface is part of an antiparallel β-sheet where no appropriate pockets are offered for binding a small molecule at high affinity^160^. Through continuous modification and optimisation, effective cyclic peptides have been designed to prevent the binding of KRAS and RAF, such as cyclorasin 9 A and cyclic peptide kD2^161,162^. Inhibition of key KRAS upstream and downstream molecules can also effectively inhibit KRAS-mediated signal transduction, such as inhibiting upstream signalling, SHP2, and inhibiting downstream signalling, the RAF-MEK-ERK pathway and the PI3K-AKT-mTOR pathway. However, due to the extensive cross-talk of KRAS-mediated signalling pathways, it is difficult to achieve effective inhibition of signalling pathways only by inhibiting a single key molecule, and joint inhibition of multiple key molecules is often required.

### Combination therapy with KRAS (G12C) inhibitors

**Fig. 6:**
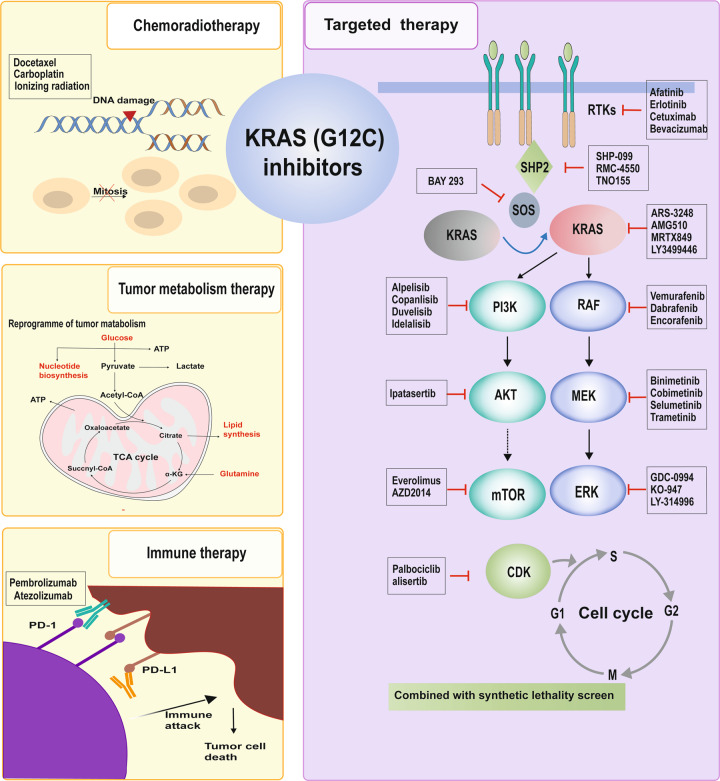
Combinational strategies for KRAS (G12C) inhibitors. The combined strategy of KRAS (G12C) inhibitors is mainly divided into four parts: combined with chemoradiotherapy, targeted therapy, immune therapy, and metabolic therapy

KRAS (G12C) inhibitors have achieved surprising results in clinical trials, and AMG510 has been successfully approved for application in the clinic, achieving a breakthrough from scratch in the development of KRAS inhibitors. However, the subsequent acquired resistance greatly limits the application of KRAS (G12C) inhibitors. Combination therapy is an important and prioritised strategy to increase efficacy, reduce toxicity and side effects, and delay the acquisition of drug resistance. Either preclinical or preliminary clinical data indicate that combinational strategies can improve the antitumour efficacy of KRAS (G12C) inhibitors. A concerning problem with combination therapy is that patients are often unable to tolerate it due to severe side effects when two or more inhibitors are applied to patients at the same time^163^. However, clinical results of KRAS (G12C) inhibitors showed that most patients tolerated treatment well without serious adverse reactions. Therefore, KRAS (G12C) inhibitors could be used as a promising class of combinational agents to achieve better efficacy (Fig. 6).

### Combined with chemoradiotherapy

Chemotherapy remains an important tumour treatment strategy for advanced cancer. However, the benefit to patients is usually limited due to severe side effects and the rapid emergence of drug resistance. The combination of conventional chemotherapy drugs and novel drugs is often considered to increase the benefit to patients. Covalent inhibitors targeting KRAS (G12C) combined with conventional chemotherapy drugs may be a potential strategy to improve efficacy. The study demonstrated that NSCLC models with KRAS mutations displayed a greater sensitivity to pemetrexed, and the expression of KRAS RNA changed after treatment with pemetrexed, leading to decreased angiogenesis, possibly because KRAS-mutant cells were more dependent on the folate metabolic pathway^164^. It is worth noting that different KRAS mutations differ in sensitivity to chemotherapy. For instance, tumour models with KRAS (G12C) responded well to taxanes and pemetrexed but responded poorly to cisplatin^164^. The combination of AMG510 and carboplatin significantly enhanced antitumour efficacy compared with AMG510 or carboplatin alone^126^. The above evidence shows that improved efficacy offers a sufficient theoretical basis for the application of a combination of KRAS (G12C) inhibitors and chemotherapeutic drugs in clinical trials. In addition, the clinical results of AMG510 and MTRX849 showed fewer adverse reactions and better tolerance, and the two resulted in more benefits and less toxicity when used in combination^131^.

In addition to the effect on chemotherapy, patients with KRAS-activated mutations often have radiation resistance and poor prognosis^165^. It has been suggested that radiation resistance is realised by EGFR-mediated chromatin condensation in KRAS-mutated lung cancers^166,167^. Radiotherapy resistance of CRC with KRAS mutations was realised by rapid upregulation of heterogeneous nuclear ribonucleoprotein KhnRNP K, and MEK inhibition effectively increased radiotherapy sensitivity of CRC^168^. Radiotherapy, as a means of cancer treatment, is widely used in many types of cancer. In radiation-naive tumours with KRAS mutations, it has been confirmed that radiotherapy combined with PD-1 antibody has an obvious synergistic effect^169^. Therefore, KRAS inhibitors in combination with radiotherapy may provide unexpected benefits to patients harbouring KRAS mutations.

## Combined with targeted therapy

### Combined with upstream molecules

#### RTKs inhibitors

The reactivation of KRAS and its downstream effectors induced by adaptive receptor tyrosine kinases (RTKs) is one of the mechanisms of drug resistance, so the combination of KRAS (G12C) inhibitors and drug-targeting RTKs may be a promising therapeutic strategy. The combined efficacy of KRAS (G12C) inhibitors and RTK inhibitors was explored in two studies^120,121^. The combined action of RTK inhibitors and ARS-853 significantly enhanced the antitumour activity in different cells, and the effects of different RTK inhibitors were different in KRAS (G12C) mutant cells, indicating the heterogeneity of the RTK action. Subsequent studies further indicated that the phosphorylation levels of several RTKs in different KRAS (G12C) models were increased and showed high heterogeneity. The synergistic effect of several RTK inhibitors and ARS-1620 showed strong antitumour activity^170^. Unfortunately, the synergistic results of these combinations are not generally effective across different KRAS (G12C) tumour models. Therefore, the efficacy of a single RTK inhibitor combined with KRAS (G12C) inhibitors may not be consistent in cancer treatment.

#### SHP2 inhibitors

Given the inconsistent effects of single RTK inhibitors, inhibiting common downstream nodes of multiple RTK signalling pathways may be broadly effective in overcoming the adaptive reactivation of KRAS^170^. Src homology region 2 domain-containing phosphatase 2 (SHP2) functions as a convergent node downstream of multiple RTKs to regulate RAS activation, which is encoded by tyrosine-protein phosphatase nonreceptor type11^171^. RTKs normally recruit the GRB2-SOS1 complex to activate RAS and do not depend on SHP2^172^. However, SHP2 is necessary for the proliferation of KRAS (G12C) mutant cancer in vivo, and adaptive reactivation of KRAS is heavily dependent on SHP2^173–175^. SHP2 inhibition has the potential to increase KRAS-GDP occupancy and suppress RAS-mediated signalling as well as adaptive signalling driving resistance to therapy^176^. For instance, the combination of ARS-1620 with SHP-099, an SHP2 inhibitor, significantly reduced tumour volume in tumour models with KRAS (G12C)^170,177^. The combination of MRTX849 with RMC-4550, another SHP2 inhibitor that also exhibited higher antitumour activity in both the sensitive and refractory MRTX849 models by further inhibiting the KRAS-mediated signalling pathway^129^. The combination of TNO155 with EGFR inhibitors, RAF inhibitors, and KRAS (G12C) inhibitors has shown synergistic effects in preclinical studies, suggesting that TNO155 effectively blocks feedback activation^178^. On the basis of these compelling preclinical data, multiple SHP2 inhibitors, such as JAB-3068, RLY-1971, and TNO155, have entered early clinical trials (NCT03518554, NCT04252339, and NCT03114319, respectively), and the combination of KRAS (G12C) and the SHP2 inhibitor is also testing in early-phase clinical trials (NCT04185883, NCT04330664).

#### SOS1 inhibitors

SOS1 is another common downstream effector of multiple RTK signalling pathways. There is increasing evidence that KRAS (G12C) rapidly cycles in the GDP or GTP binding status rather than being continuously active in the GTP-KRAS status, resulting in abnormal activation of downstream signals. This indicates that KRAS (G12C) is still sensitive to GEF (such as SOS1) regulation. A synergistic effect of BAY293, a potent SOS1 inhibitor, with ARS-853 has been reported because BAY293 is capable of disrupting the KRAS-SOS1 interaction to inhibit the activation of KRAS^179^.

### Combined with downstream molecules

KRAS is cross-linked with various signalling pathways and plays a pivotal role in cell survival and proliferation. In turn, downstream effectors influence KRAS function to different degrees. For instance, ERK3 activity is required for KRAS-driven tumorigenesis in vitro, and ERK3 deficiency inhibits the oncogenic growth of KRAS (G12C)-mutant NSCLC in vivo, indicating the application of ERK3 inhibitors in KRAS (G12C)-driven tumours^180^. Recent studies have found that BRAF/ERK inhibitors can release the feedback inhibition of upstream RTK and RAS signals, and the liberation of RTK and RAS signals can facilitate other signalling pathways, such as the PI3K/AKT/mTORC1 pathway, which improves cell growth and proliferation^181^. The combination of a PI3K inhibitor and a KRAS (G12C) inhibitor showed obvious synergistic effects in various models. In addition, targeting ATK or mTOR, important PI3K downstream effectors, has been proven to be effective when used in combination with KRAS (G12C) inhibitors^126,129,182^. For instance, the combined use of mTOR inhibitors, IGF1R inhibitors, and ARS-1620 significantly enhanced tumour regression with low toxicity compared to combined MEK inhibitors or ARS-1620 alone in a series of KRAS-driven mouse lung cancer models^183^.

### Combined with cell cycle checkpoint

Excessive proliferation is a common feature of tumour cells, and CDK4/6 is a key regulator of the cell cycle, playing a key role in the G1-to-S-phase transition. CDK4/6 can be affected by KRAS through multiple signalling pathways, such as the RAF-MEk-ERK and PI3K-Akt pathways. Studies have shown that the CDK4/6 inhibitor palbociclib enhances the overall effect of a KRAS inhibitor (ARS-1620). Similarly, another study indicated that MRTX849 and palbociclib, in several MRTX849-refractory models, exhibited significant general tumour regression, which may be due to increased inhibition of the retinoblastoma protein/E2F transcription factor pathway^129^. In addition to CDK, aurora kinase A (AURKA) plays an important role in the cell cycle by ensuring the proper separation of chromosomes and the smooth completion of cytokinesis during mitosis. ARS-1620 was observed to have a significant synergistic effect with alisertib, a specific AURKA inhibitor, for the treatment of ARS-1620-refractory KRAS (G12C) mutant cancer. In terms of mechanism, targeting AURKA not only affects the normal proliferation division of cells but also disrupts the stable interaction between KRAS and c-RAF, resulting in difficulty in RAF activation^177^. Several clinical trials are currently underway in which combinations of CDK4/6 inhibitors have been added (Table 3).

### Combined with synthetic lethality screen

Compared with wild-type KRAS cells, mutant KRAS-driven tumour cells may be more dependent on certain genes that are necessary for the maintenance of the KRAS-driven cellular state. Targeting these vulnerable genes in synthetic lethality screens could serve as an alternative approach, called synthetic lethality screens. Some large-scale genetic screening has been performed to find vulnerable genes that are uniquely essential to KRAS-mutant tumours^184–186^. The study found that in addition to KRAS itself, the critical genes for Kras mutation were RAF1 (encoding CRAF) and SHOC2^187^. Genetic deletion of CRAF significantly reduced tumour size in a KRAS (G12V) mouse model, which was independent of the function of CRAF kinase^188^. SHOC2 enhances RAF dimerization and is necessary for maximum ERK activity. Ablation of SHOC2 reduced the growth of KRAS-mutant NSCLC cells and sensitised the cells to MEK inhibition^189^. Another experiment showed strong enrichment of genes with mitotic functions in a genome-wide RNAi screen of KRAS-mutant cells^186^. In addition, genome-wide CRISPR/Cas9-based genetic screening has been widely used to find ideal synthetic lethal targets in KRAS-mutated tumours^190^. Unfortunately, the degree of overlap between these screen results is low, except for the proteasome system, and has not yet led to successful clinical approaches. However, the successful development of KRAS (G12C) inhibitors provides a new direction for KRAS mutation-related synthetic lethality. One study proposed that the bypass pathways (defined as collateral dependencies (CDs)) that maintain cancer cell survival may be different from those dependent on the excessive activation of KRAS signalling when acute deprivation drives oncogene activity with KRAS (G12C) inhibitors and target CDs will promote the response to KRASG12C inhibitors^191^. At the same time, two classes of combination strategies targeting CDs are proposed to either strengthen KRAS (G12C) target engagement (namely, EGFR, FGFR, or SHP2 inhibitors) or independently restrain persistent survival pathways (namely, PI3K, or CDK4/6 inhibitors)^191^.

In general, synergistically targeting upstream molecules of KRAS is considered to offer advantages over synergistically targeting downstream molecules of KRAS. Synergistic targeting of upstream molecules can effectively inhibit multiple downstream pathways, avoiding the activation of other parallel signalling pathways that can induce tumour growth. However, the identification of appropriate upstream targets for KRAS is challenging and risky because cotargeting upstream targets may bring more side effects^192^. Synthetic lethal approaches using genome-wide screening are also promising, but more detailed screening conditions are needed to address complex tumour types. In addition, effective synthetic lethal targets may be defined as subsets of tumours. For instance, KRAS-mutated tumours with KEAP1 mutations and activation of NRF2 antioxidant programmes make them more susceptible to interference with the glutathione pathway^193^.

### Combined with immune therapy

In recent decades, great progress has been made in immunotherapy for advanced cancer^194^. There has been substantial clinical evidence that the survival of patients with advanced cancer is improved by immune checkpoint inhibitors (ICIs), especially by blocking the immune checkpoint axis involved in programmed cell death 1 (PD-1) and programmed death ligand 1 (PD-L1)^195,196^. PD-1/PD-L1 inhibitors such as durvalumab, nivolumab, pembrolizumab, and atezolizumab have entered the clinic as first-line or second-line therapy for advanced NSCLC^196–198^. However, clinical data from anti-PD therapy indicated that only approximately 15–25% of patients with NSCLC responded to ICIs^199^. Among various drug resistance factors, the TME plays a significant role in the host response to immunotherapy^200^. Targeted therapy usually has a profound immunomodulatory effect on the TME, so targeted therapy combined with immunotherapy should be a strong combinational therapeutic strategy^201,202^.

Novel KRAS (G12C) inhibitors induce a shift of the TME from immunosuppressive to immunoreactive. Increasing evidence suggests that KRAS mutation preferentially induces an immunosuppressive TME by promoting the expression of immunomodulatory factors of tumour cells, such as transforming growth factor-β, interleukin-6, and interleukin-10^203^. KRAS inhibition might be able to block the effect. A relative study has shown that the infiltration of CD8 + T cells, macrophages and dendritic cells in the TME is significantly increased after AMG510 treatment, including CD103 + cross-presenting dendritic cells, which are necessary for T cell priming, activation, and recruitment^126^. In general, AMG510 therapy contributes to the formation of a proinflammatory microenvironment and enhances immunosurveillance. Similarly, MRTX849 treatment decreased intratumoural immunosuppressive myeloid-derived suppressor cells and M2-polarised macrophages and increased immune-promoting M1-polarised macrophages, dendritic cells, and CD4 + and NKT cells in KRAS (G12C) tumours by altering the expression of tumour RNA and protein implicated in the presentation of tumour antigens or mediating an immunosuppressive TME^204^. These results agree with a previous study showing that patients with KRAS mutations usually have an inflammatory TME and higher tumour immunogenicity, leading to a better response to PD-1/PD-L1 inhibitors^205^. Notably, the durable antitumour response to KRAS (G12C) inhibitors depends on the engagement of the immune system. Treatment with high doses of AMG510 displayed sustained tumour regression in immunocompetent mouse models, whereas tumours recovered rapidly after a short response in immunocompromised mouse models^126^. These studies suggest that specific inhibitors targeting KRAS (G12C) in tumour cells profitably result in a turn from an immunosuppressive to an immunocompetent TME; thus, the combination of KRAS (G12C) inhibitors and immune checkpoint inhibitors is a promising reciprocal strategy for patients with KRAS (G12C) mutant NSCLC.

### Combined with tumour metabolism therapy

One of the hallmarks of cancer is the reprogramming of cellular metabolism, in which the tumour efficiently uses substances in the environment to provide more energy and biomacromolecules to meet the nutritional needs of the tumour’s uncontrolled proliferation^206^. Many studies have shown that KRAS mutations can lead to tumour-specific metabolic changes, thereby regulating oncogenic signalling networks and promoting tumour progression^207,208^. Mutant KRAS upregulates the expression of the GLUT1 glucose transporter to promote glucose uptake and glycolytic rate-limiting enzymes hexokinase 1 and 2 to increase glycolytic activity^209,210^. Mutant KRAS also promotes the generation of precursors of many biomacromolecules, such as the precursor for lipid and protein glycosylation through the hexosamine biosynthesis pathway^211^ and the backbone for nucleic acid production^212^. In addition to affecting glycolysis, mutant KRAS promotes tumorigenesis and development by regulating glutamine decomposition, lipid metabolism and fatty acid biosynthesis^213–216^. In addition, mutated KRAS meets its own needs of high nutrition and rapid metabolism through high levels of autophagy and macropinocytosis^217–219^.

The close association between the KRAS signalling and cellular metabolic remodelling makes it possible to exploit KRAS-associated metabolic fragility to treat KRAS-driven cancers^220^. As expected, targeted metabolic enzymes have been shown to be effective in some KRAS-mutant cancer cell lines and in mouse models^221,222^. Some clinical trials are underway targeting the metabolic pathways of KRAS mutant cancers, including strategies to combat glutamine breakdown and autophagy. Indeed, synergistic effects of several metabolic pathway inhibitors in combination with KRAS (G12C) inhibitors, such as the mTOR inhibitors and MRTX849, have been tested in preclinical settings with encouraging results^223^. However, much work remains to be done to explore the therapeutic potential of targeting metabolic changes in KRAS-driven cancers and synergistic effects with KRAS inhibitors^224^.

### The acquired resistance mechanism of covalent KRAS (G12C) inhibitors

**Fig. 7:**
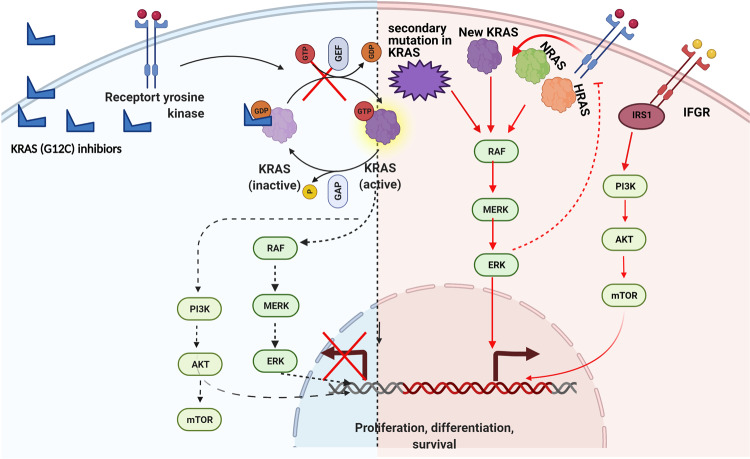
Acquired resistance mechanism of covalent KRAS (G12C) inhibitors. The figure on the left blue background represents cells that are sensitive to KRAS (G12C) inhibitors, while the figure on right red background represents cells that are resistant to inhibitors. The black dotted line represents inhibition of KRAS signalling in the presence of KRAS (G12C) inhibitors. The solid red lines represent the resistance mechanisms identified after the use of KRAS (G12C) inhibitors, including the relief of ERK-mediated feedback inhibition, which reactivates the MAPK pathway by wild-type RAS (NRAS and HRAS) or new synthetic KRAS (G12C); the activation of other bypasses, such as PI3K activation by the IGFR–IRS1 pathway and secondary or additional mutations in KRAS. This figure generated from BioRender

Despite acquiring promising preliminary clinical results from KRAS (G12C) inhibitors, it should be cautioned that complete responses are rare in clinical trials^225,226^. The diversity of tumour status and the sensitivity to drugs in different populations reasonably influence the drug effect because of various intrinsic factors, including dependency on KRAS-mediated signalling and concurrent genetic alterations^227^. Simultaneously, rapidly acquired drug resistance after promising initial responses is a very common challenge in cancer monotherapy targeting oncogenic driving enzymes. It is conceivable that there is a high possibility of acquired resistance after treatment with KRAS (G12C) inhibitors^228^. Several adaptive resistance mechanisms have been proposed, including the release of ERK-mediated feedback inhibition, activation of other bypasses, secondary KRAS mutations, and multiple resistance mechanisms that occur simultaneously (Fig. 7).

Experience gained from RAF and MEK inhibitors that have been applied in the clinic and extensively studied in their resistance mechanisms could provide important insights into the resistance mechanisms to KRAS (G12C) inhibitors^229^. For instance, an increased level of ERK phosphorylation after long-term treatment with RAF or MEK inhibitors was reported, which may be caused by the amplification of upstream drivers such as RTKs^230^. Similarly, after ARS-1620 treatment for a period of time, the recovery of ERK pathway flux was identified in vivo^124^. These results strongly imply that treatment with KRAS (G12C) inhibitors triggers the release of ERK-mediated feedback inhibition, which reactivates KRAS signalling in turn to confer therapeutic resistance. The work of Ryan and colleagues supported this view and proposed the mechanism of drug resistance in which wild-type RAS, which could not be specifically inhibited by KRAS (G12C) inhibitors, rapidly activated the adaptive KRAS feedback pathway mediated by PTK after ARS-1620 and AMG510 treatment in vitro^170^. Furthermore, no prominent RTK was found to mediate this process in all KRAS (G12C) tumour models, suggesting that this feedback may be comodulated by multiple RTKs. In an in vivo study, KRAS (G12C) colorectal cancer models had higher basal receptor tyrosine kinase (RTK) activation, especially EGFR signalling, than NSCLC cell lines. Therefore, KRAS (G12C) inhibition induced a higher rebound of phosphorylated ERK than NSCLC cells, which resulted in a generally poorer response to KRAS (G12C) inhibitors in CRC patients than in NSCLC^231^. Another in vitro study also supported this view, but a diverse mechanistic interpretation suggested that after treatment with KRAS (G12C) inhibitors, some cancer cells were in a stationary state with low KRAS activity, while others were equipped to resume proliferation due to the synthesis of new KRAS (G12C)^177,232^. In addition, it was reported that GEFR promoted the activation of newly synthesised KRAS, and aurora kinase A (AURKA) avoided drug-induced tumour cells by interacting with KRAS (G12C) and the downstream protein c-RAF^177^.

Epithelial-to-mesenchymal transition (EMT), another reason for resistance, was proposed in the presence of KRAS (G12C) inhibitors, which activated the PI3K pathway to lead to endogenous and acquired drug resistance. In EMT-induced cells, Adachi and colleagues demonstrated that PI3K remained activated in the presence of KRAS (G12C) inhibitors and was dominantly regulated by the IGFR–IRS1 pathway^233^. Another in vitro study supported this view and proposed that epithelial cells compensated through the ERBB2/3 signalling pathway, while mesenchymal cells exhibited high basal and feedback FGFR activation following inhibition of ERK and AKT signalling by ARS-1620 treatment^234^. In addition, considering that KRAS (G12C) covalent inhibitors inactivate KRAS (G12C) by locking them in GDP binding status, KRAS (G12C) could have a secondary mutation that led to the loss of GTPase activity or decreased affinity with GDP^235^. A clinical report described a patient with KRAS (G12C) NSCLC who developed polyclonal acquired resistance to MRTX849 and showed 10 heterogeneous resistance changes in 4 genes, including KRAS, NRAS, BRAF, and MAP2K1. Of note, the researchers identified an important mutation in KRAS (Y96D) that affected the Switch-II pocket, resulting in resistance to all current KRAS (G12C) inhibitors^236^. At the same time, researchers also reported a novel, functionally distinct tricomplex KRAS (G12C) active-state inhibitor RM-018, which can inhibit KRAS (G12C/Y96D) in vitro^237^. Evidence from in vitro studies confirms this conclusion, and it is clear that either AMG510 or MRTX849 results in a high proportion of KRAS secondary mutations, and 12 different secondary KRAS mutations are found. Different KRAS secondary mutations have different resistances to different drugs. For example, G13D, R68M, A59S and A59T mutations were resistant to AMG510 but sensitive to MRTX849, while the Q99L mutation was resistant to MRTX849 and remained sensitive to AMG510^238^. The results of a clinical cohort study (NCT03785249) further indicate that the mechanisms of acquired resistance to KRAS (G12C) inhibitors are quite complex, with multiple mechanisms occurring simultaneously in a single patient. Required KRAS alterations included G12D/R/V/W, G13D, Q61H, R68S, H95D/Q/R, Y96C and high-level amplification of the KRASG12C allele. Acquired bypass mechanisms of resistance included MET amplification, activating mutations in NRAS, BRAF, MAP2K1, and RET, oncogenic fusions involving ALK, RET, BRAF, RAF1, and FGFR3, and loss-of-function mutations in NF1 and PTEN^239^.

These studies partly explained why patients responded partially but not completely to treatment with KRAS (G12C) inhibitors. Furthermore, resistance could potentially occur via other undiscovered mechanisms, even multiple mechanisms, emphasizing the need to further investigate resistance mechanisms to select optimal treatment regimens for best benefits.

## Concluding remarks and future perspectives

Targeting KRAS is an attractive strategy because of the high prevalence of KRAS mutations and its importance in initiating and sustaining tumour growth. Because of its own characteristics, targeting KRAS directly was once thought impossible. With continuous active exploration, there have been several novel insights to better understand KRAS mutations, promoting the development of drugs targeting KRAS. Progress has been made in targeting KRAS, especially targeting KRAS (G12C). New techniques, such as NMR-based fragment screening, tethering, and in silico drug design, have been used to discover novel agents that bind directly to KRAS, although there is a long way off to achieve targeted KRAS. Unconventional approaches of peptides and proteins are also promising but face delivery-related challenges. A class of covalently specific small molecules that bind KRAS (G12C), such as AMG510 and MRTX849, have been identified and have shown promising results in clinical trials. However, there are also some problems to be solved for its subsequent development, including the evaluation of clinical safety in a larger cohort, the optimisation of clinical efficacy and the overcoming of drug resistance. The response to KRAS (G12C) inhibitors in patients is diverse, implicating the existence of intrinsic resistance. Further exploration of intrinsic resistance should be conducted to identify biomarkers that indicate the appropriate population and tumour type in the clinic. Meanwhile, the common challenge for targeted drugs is the emergence of acquired resistance. Further investigation is clearly warranted to comprehensively elucidate the mechanisms of acquired resistance to obtain optimal treatment options.
